# Combination of biological aging and genetic risk helps identify at-risk population of thoracic aortic aneurysms: insight from a prospective cohort study of UK Biobank

**DOI:** 10.1097/JS9.0000000000002898

**Published:** 2025-06-30

**Authors:** Jiatang Xu, Zhensheng Hu, Hongze Liu, Yangfan Su, Runnan Shen, Chaoyu Xie, Yi Zhou, Kai Huang

**Affiliations:** a Zhongshan School of Medicine, Sun Yat-sen University, Guangzhou, China; b Department of Cardiovascular Surgery, The First Affiliated Hospital of Sun Yat-sen University, Sun Yat-sen University, Guangzhou, China; c Department of Vascular Surgery, Sun Yat-sen Memorial Hospital Sun Yat-sen University, Guangzhou, China; d Department of Urology Surgery, Sun Yat-sen Memorial Hospital, Sun Yat-sen University, Guangzhou, China

**Keywords:** PhenoAgeAccel, polygenic risk score, risk stratification, thoracic aortic aneurysms

## Abstract

**Background:**

Both age and genetic risk are associated with risk of thoracic aortic aneurysms (TAA). This study seeks to explore the association between phenotypic age acceleration (PhenoAgeAccel), a novel biomarker of aging, and risk of TAA, as well as to conduct risk stratification for TAA based on PhenoAgeAccel and genetic risk.

**Methods:**

A total of 406 750 participants from the prospective cohort of UK Biobank were included. PhenoAgeAccel was calculated based on chronological age and 9 biomarkers. Associations between PhenoAgeAccel, genetic risk and incident TAA were explored based on cox proportional hazards models. Additionally, mediation analyses were conducted to explore whether PhenoAgeAccel mediated the pathogenic process of risk factors for TAA.

**Results:**

PhenoAgeAccel was significantly associated with increased risk of TAA (hazard ratio [HR]: 1.31; 95% confidence intervals [CI]: 1.15–1.48). Compared to biologically younger participants with low genetic risk, biologically older participants with high genetic risk had a 3.73 (95% CI: 2.70–5.16) folds risk of TAA. PhenoAgeAccel exhibited a significant additive interaction with genetic susceptibility. Mediation analyses revealed that PhenoAgeAccel mediates the association between various risk factors and the progression of TAA.

**Conclusion:**

PhenoAgeAccel has the potential to serve as a novel aging biomarker for identifying high-risk populations of TAA. The combination of PhenoAgeAccel and genetic risk can further improve TAA risk stratification, thereby informing the formulation of screening strategy and primary prevention for TAA.

## Background

Thoracic aorta aneurysms (TAA) represent a type of disease with significant threat and mortality[1]. TAA can progress to dissection and rupture, both of which pose the high risk of sudden cardiac death^[2,3]^. Early screening and intervention to mitigate progression and avert fatal outcomes are crucial in the management of TAA[4]. Regrettably, the enlarged thoracic aorta typically presents asymptomatically prior to rupture or dissection, while the implementation of common screening for the thoracic aorta is still not widespread, leading to diagnosis of TAA often not timely^[4,5]^. Consequently, it is essential to explore indicators for effectively quantifying the risk of TAA and identifying at-risk population.

**HIGHLIGHTS**

- Innovative aging biomarker and TAA risk: Based on a large prospective cohort from the UK Biobank, we constructed PhenoAgeAccel to quantify individual accelerated aging and explored the association between accelerated aging, and incident thoracic aortic aneurysms (TAA).
- Combining biological aging and genetic risk: PhenoAgeAccel demonstrated a significant additive interaction with genetic risk for TAA. Individuals with high genetic risk and accelerated aging faced the highest risk of TAA, with a HR of 3.73 (95% CI: 2.70–5.16).
- Explored the potential pathogenic mechanism: Mediation analyses showed TAA risk factors, such as hypertension and hyperlipidemia, may promote the occurrence of TAA by accelerating aging.
- Guidance for prevention strategies: Our findings promote the integration of genetic and biological aging biomarkers into the screening strategies and personalized prevention for TAA.

Age has been recognized as an independent predictor of TAA, while hypertension, an age-related disease, is recognized as the most significant risk factor for TAA^[6,7]^. However, individuals with the same chronological age may exhibit different rates of aging, leading to varying disease risks. This underscores the importance of exploring the role of biological age in the pathogenesis of TAA. Phenotypic age acceleration (PhenoAgeAccel) is a novel biological aging metric based on multiple systemic indicators, capturing the disparity between individuals’ biological and chronological age[8]. Single nucleotide polymorphisms (SNPs) associated with PhenoAgeAccel have been implicated in various cardiovascular diseases and TAA risk factors, such as atrial fibrillation and blood pressure[9]. Therefore, we would like to explore whether PhenoAgeAccel can assist in identifying individuals at high risk of TAA.

TAA demonstrates genetic susceptibility, with heritability estimated at 57%[10]. Related studies have successfully identified multiple SNPs associated with TAA, and further constructed polygenic risk scores (PRS) to quantify the risk of TAA^[11,12]^. Previous studies have demonstrated that PhenoAgeAccel can interact with genetic susceptibility and jointly exert an influence on individuals’ disease risk[13]. Based on the data from the UK Biobank, we conducted this study to investigate the association between PhenoAgeAccel, genetic risk and incident TAA, thereby informing the formulation of screening strategy and primary prevention for TAA.

## Methods

### Study population

Our research population is derived from the UK Biobank. Between 2006 and 2010, the UK Biobank recruited over 500 000 participants to build this prospective cohort[14]. Participants were enrolled across 22 assessment centers nationwide, where they provided baseline data, biological samples, and written informed consent. Inclusion criteria for this study were age between 40 and 70 years and provision of a written informed consent, while exclusion criteria were prior diagnosis of TAA before recruitment, and with substandard genetic data. Substandard genetic data were defined by autosomal missing rate that exceeds 0.02, identification of outliers associated with missingness or heterozygosity, inclusion of individuals outside a maximal set of unrelated participants, sex-inconsistent and lack of genetic data.

We conducted this study in line with the updated STROCSS 2025 criteria[15]. The UK Biobank involved human participants, and received ethical approval from the North West Multicenter Research Ethics Committee (MREC) under reference number 21/NW/0157. This research was conducted as part of resource project 100739 from UK Biobank.

### Ascertainment of outcome

The primary outcome of this study is incident TAA during the follow-up. The diagnosis of incident TAA was established based on the International Classification of Diseases version 10 (ICD-10) codes along with the Office of Population Censuses and Surveys Classification of Interventions and Procedures version 4 (OPCS-4) codes. Cutoff time was set as the earliest date among TAA diagnosis, death, or last follow-up. The last follow-up dates varied by region: 31 October 2022, for England; 31 May 2022, for Wales; and 31 August 2022, for Scotland. Details of incident TAA diagnosis were shown in Supplemental Digital Content, Table 1 (available at: http://links.lww.com/JS9/E496).

### Phenotypic age and PhenoAgeAccel calculation

Plasma biomarkers utilized in the construction of phenotypic age include creatinine, C-reactive protein, glucose, albumin, lymphocyte percent, alkaline phosphatase, white blood cell count, mean cell volume and red blood cell distribution width. And the resulting final equation for calculating phenotypic age is as follows^[8,16]^:

*xb* = −19.907 − 0.0336 × albumin + 0.0095 × creatinine + 0.1953 × glucose + 0.0954 × ln(C-reactive protein) − 0.0120 × lymphocyte percentage + 0.0268 × mean corpuscular volume + 0.3306 × red blood cell distribution width + 0.00188 × alkaline phosphatase + 0.0554 × white blood cell count + 0.0804 × chronological age

$$MortalityRisk=1-exp\left(\frac{-1.51714\times exp\left(xb\right)}{0.0076927}\right)$$

$$\;\;PhenotypicA\geq 141.50225\;+\;\frac{ln[-0.00553\times ln(1-MortalityRisk)]}{0.090165}$$

from the linear regression between phenotypic and chronological age, reflecting whether an individual is physiologically older (PhenoAgeAccel > 0) or younger (PhenoAgeAccel < 0) than their chronological age. Plasma biomarkers for the construction of phenotypic age were collected at recruitment and detailed in Supplemental Digital Content, Table 2 (available at: http://links.lww.com/JS9/E496).

### PRS calculation

Heritable variants linked to TAA were utilized in the PRS calculation of TAA. Building on SNPs associated with TAA identified in prior studies, two PRSs were applied to assess participants’ genetic susceptibility of TAA, named PRS-89 and PRS-56, respectively including 89 and 56 TAA-related SNPs^[11,12]^. Methodologies for SNP genotyping and imputation within the UK Biobank cohort have been comprehensively detailed in a prior publication[14]. Employing a linkage disequilibrium (LD) clumping cutoff of r2 < 0.05 and conducting conditional analyses, we implemented two polygenic risk scores (PRSs) based on the SNP data from the referenced studies. Each SNP was encoded as 0, 1, or 2, representing the count of risk alleles present. The weighted PRSs were calculated using the PLINK “–score” command, based on the following equation:

$$PRS=\left(\beta_{1}\times SNP_{1}+\beta_{2}\times SNP_{2}+\cdots +\beta_{i}\times SNP_{i}+\cdots +\beta_{n}\times SNP_{n}\right)$$

Where n represents the cumulative count of SNPs, and βi denotes the log odds ratio (OR) per allele for TAA linked to each SNPi. The effect size estimates of βi were derived from previous studies.

Genetic susceptibility of participants was categorized based on their PRS distribution into three categories: low risk (the lowest 20%), median risk (20–80%), and high risk (the top 20%)^[13,17]^. Details of the SNPs employed in PRS calculation were shown in Supplemental Digital Content, Tables 3 and 4 (available at: http://links.lww.com/JS9/E496).

### Ascertainment of covariates

To mitigate potential bias of results caused by various confounding factors, we incorporated participants’ body composition, lifestyle habits, and comorbidities at baseline into the comprehensive analysis. Body mass index (BMI) was derived from height and weight measurements to indicate an individual’s level of obesity. Educational background of participants was divided into the “College or University degree” category and the “Other” category based on the attainment of higher education. The Townsend Deprivation Index (TWI) served as a metric for assessing the level of material deprivation within populations and was readily accessible from the UK Biobank[18]. Smoking status and alcohol consumption frequency, recognized as prevalent risk factors for various diseases, were also incorporated into the analysis of this study. Additionally, comorbidities such as diabetes, hypertension, hyperlipidemia, and malignancies were recorded at baseline. Ascertainment of covariates were detailed in Supplemental Digital Content, Table 5 (available at: http://links.lww.com/JS9/E496).

### Statistical analyses

For continuous variables, missing values were imputed using average values by sex, while categorical variables with missing data were categorized as “unknown.” Additionally, to avoid errors and biases in calculation of PhenoAgeAccel due to extreme values of plasma markers, we set the first and last 1% of data for each variable as the 1st and 99th percentiles, respectively. Details of missing value were shown in Supplemental Digital Content, Table 6 (available at: http://links.lww.com/JS9/E496).

Participants with positive PhenoAgeAccel value were classified as biologically older, whereas participants with a negative PhenoAgeAccel value were classified as biological younger. Cumulative risk curves for participants with varying PhenoAgeAccel levels were constructed and log-rank test was performed to assess differences. Testing proportional hazards assumption by the using of Schoenfeld residuals[19]. Cox proportional hazards models were conducted to assess the association between PhenoAgeAccel, PRS, and incident TAA. Moreover, we generated restricted cubic spline (RCS) curves to investigate the potential nonlinear relationship between PhenoAgeAccel and TAA risk.

Sensitivity analyses were performed to ensure the robustness of our study findings. First, we excluded individuals who experienced a TAA outcome within 2 years to mitigate potential reverse causality. Furthermore, we implemented propensity score matching (PSM) to match the biologically older participants and biologically younger participants, at a ratio of 1:1 to minimize bias introduced by confounding factors. Last, individuals with missing values for covariates were excluded to reduce the bias introduced by data imputation.

Relative excess risk (RERI) and attributable proportion (AP) were calculated based on Delta method to investigate the potential additive interaction between PhenoAgeAccel and PRS. RERI quantified the increase in risk of TAA resulting from the joint effect of PhenoAgeAccel and genetic risk, while AP represented the percentage of the total TAA risk that RERI accounts for. To investigate whether PhenoAgeAccel serves as a mediator between TAA-related factors and TAA, mediation analyses were conducted with PhenoAgeAccel as the mediator factor. Additive interaction and mediation analyses were conducted by the using of the R packages “interaction” and “Mediation.”

TAA prediction models were constructed based on cox regression models, and time-dependent receiver operating characteristic curves were used to verify whether PhenoAgeAccel and genetic risk can enhance the prediction of TAA. Using the time sequence of recruitment, the train cohort of the model was composed of the first 75% of the participants (305 062), while the subsequent 25% (101 688) were designated for the test cohort. All analyses were done in RStudio version 4.2.1, and a two-sided *P* value of <0.05 was considered to be statistically significant.

## Result

### Participants

**Figure 1.**
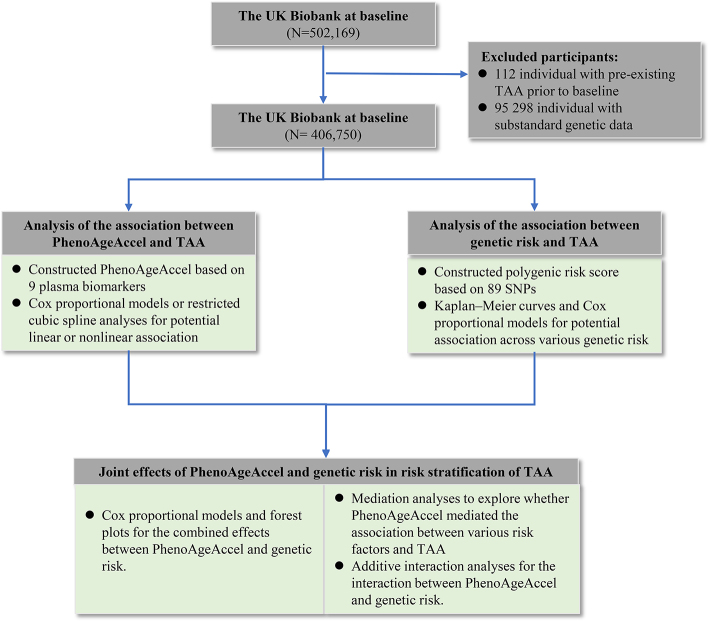
Design and work flow of this study.

UKB: UK Biobank; TAA: thoracic aortic aneurysms; PhenoAgeAccel: phenotypic age acceleration; SNPs: single nucleotide polymorphisms

A total of 406 750 participants were ultimately included in this study, as shown in Figure 1. During a median follow-up period of 13.63 years, 1055 participants (0.26%) were reported incident TAA based on ICD-10 and OPCS-4. Of all participants, 184 579 (45.38%) were classified into the biologically older, while 222 171 (54.62%) were classified into the biologically younger. Although no significant difference in chronological age was observed, the median phenotypic age between the two groups differed by 7.62 years. The proportion of participants who developed incident TAA was significantly higher in the biologically older group. Baseline data of the included population were presented in Table 1.

**Table 1:** Baseline characteristics of participants

	Total	Biologically older	Biologically younger	*P* value
Participants	406 750	184 579	222 171	
Chronological age, years	56.47 ± 8.10	56.45 ± 8.26	56.49 ± 7.95	0.141
Phenotypic age	53.22 [45.65, 59.77]	57.81 [49.63, 64.12]	50.22 [42.91, 56.12]	<0.001
PhenoAgeAccel, years	−0.51 [−3.34, 2.65]	3.04 [1.35, 5.72]	−3.05 [−5.01, −1.49]	<0.001
Sex, male	187 317 (46.05%)	101 280 (54.87%)	86 037 (38.73%)	<0.001
BMI, kg · m^−2^	26.80 [24.20, 29.80]	27.84 [25.10, 31.20]	26.00 [23.60, 28.60]	<0.001
Townsend deprivation index	−2.14 [−3.65, 0.55]	−1.87 [−3.51, 1.07]	−2.33 [−3.75, 0.12]	<0.001
History of hypertension	222 880 (54.80%)	110 087(59.64%)	112 793(50.77%)	<0.001
History of diabetes	24 156 (5.94%)	18 343 (9.94%)	5813 (2.62%)	<0.001
History of hyperlipidemia	71 050 (17.47%)	38 770 (21.00%)	32 280 (14.53%)	<0.001
Malignant tumor	33 521 (8.24%)	15 661 (8.48%)	17 860 (8.04%)	<0.001
Ethnicity				<0.001
White	380 680 (93.59%)	171 611 (92.97%)	209 069 (94.10%)	
Mixed	2519 (0.62%)	1250 (0.68%)	1269 (0.57%)	
Asian	8553 (2.10%)	4258 (2.31%)	4295 (1.93%)	
Black	6964 (1.71%)	3878 (2.10%)	3086 (1.39%)	
Other	5472 (1.35%)	2263 (1.22%)	3209 (1.44%)	
Unknown	2562 (0.63%)	1319 (0.71%)	1243 (0.56%)	
Education background				<0.001
College or university	135 321 (33.27%)	54 951 (29.77%)	80 370 (36.18%)	
Other	266 091 (65.42%)	126 779 (68.69%)	139 312 (62.70%)	
Unknown	5338 (1.31%)	2849 (1.54%)	2489 (1.12%)	
Alcohol intake				<0.001
More than once a week	280 934 (69.07%)	122 709 (66.44%)	158 225 (71.22%)	
Less than once a week	124 850 (30.69%)	61 319 (33.20%)	63 531 (28.60%)	
Unknown	966 (0.24%)	551 (0.30%)	415 (0.19%)	
Smoking status				<0.001
Current or previous	182 622 (44.90%)	90 710 (49.14%)	91 912 (41.37%)	
Never	222 006 (54.58%)	92 715 (50.23%)	129 291 (58.19%)	
Unknown	2122 (0.52%)	1154 (0.63%)	968 (0.44%)	

PhenoAgeAccel: phenotypic age acceleration; BMI: body mass index; TAA: thoracic aorta aneurysm; Categorical variables were expressed as percentages, compared with chi-square tests, continuous variables were represented by the median with interquartile range (IQR) or mean with standard (SD), and compared using the Mann-Whitney U test or t test. A two-sided *P* < 0.050 was considered statistically significant. Biologically older means participants with positive PhenoAgeAccel value, while biologically younger means participants with negative PhenoAgeAccel value.

### PhenoAgeAccel and risk of TAA

**Figure 2.**
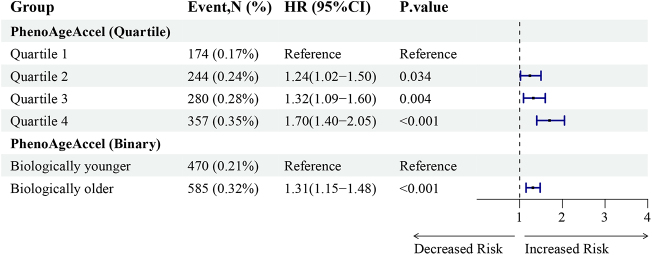
Association between PhenoAgeAccel and TAA.

**Figure 3.**
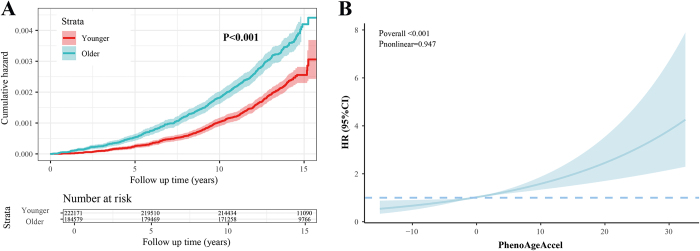
Cumulative risk and restricted cubic spline curves to assess the risk of TAA across different PhenoAgeAccel levels. Cumulative risk curves for TAA risk across different PhenoAgeAccel levels. (A) Cumulative risk curves for TAA risk across different PhenoAgeAccel levels. (B) Linear association between PhenoAgeAccel and the risk of TAA. HR: hazard ratio; TAA: thoracic aortic aneurysms; PhenoAgeAccel: phenotypic age acceleration; Restricted cubic spline curves adjusting for chronological age, sex, ethnicity, education, body mass index, Townsend deprivation index, smoking status, alcohol intake frequency, history diabetes, hypertension, hyperlipidemia and malignancy.

Participants with incident TAA during follow-up generally exhibited higher PhenoAgeAccel (Supplemental Digital Content, Figure 1, available at: http://links.lww.com/JS9/E496). As shown in Figure 2, biologically older individuals faced a significantly increased risk of TAA (HR: 1.31; 95% CI: 1.15–1.48; *P* < 0.001). Moreover, our investigation revealed a significant increase in risk of TAA from participants in the 1st to the 4th quartile of PhenoAgeAccel. We further constructed the cumulative risk and RCS curve to assess the relationship between different levels of PhenoAgeAccel and risk of TAA (Fig. 3). And results showed a positive, linear association between increasing PhenoAgeAccel and elevated TAA risk (*P* for overall < 0.001; *P* for nonlinear = 0.947).

PhenoAgeAccel: phenotypic age acceleration; TAA: thoracic aorta aneurysm;Model was adjusted for chronological age, sex, ethnicity, education, body mass index, Townsend deprivation index, smoking status, alcohol intake frequency, history diabetes, hypertension, hyperlipidemia, and malignancy

### Stratified and sensitivity analyses

To assess the robustness of the association between PhenoAgeAccel and TAA, we conducted further stratified and sensitivity analyses. Given that the UK Biobank comprises a generally healthy population with white ancestry, we focused on exploring the association between PhenoAgeAccel and incident TAA across different health conditions and ethnicities in stratified analyses. Specifically, we analyzed this association in groups with varying baseline ages, ethnicities (white and other), the presence of major risk factors for TAA (hypertension and hyperlipidemia) at baseline, and the presence of cardiovascular diseases at baseline. The results demonstrated that association between PhenoAgeAccel and TAA remained significant across different subgroups (Supplemental Digital Content, Table 7, available at: http://links.lww.com/JS9/E496). Additionally, cumulative risk curve revealed that biologically older participants exhibited a significantly elevated risk of TAA across all subgroups (Supplemental Digital Content, Figures 2 and 3, available at: http://links.lww.com/JS9/E496). In the sensitivity analyses, we excluded participants who reported incident TAA within 2 years, and the results indicated that the association between PhenoAgeAccel and incident TAA remained significant. Moreover, similar results were observed after adjusting for confounding factors through PSM and excluded individuals with missing values for covariates (Supplemental Digital Content, Figure 4, available at: http://links.lww.com/JS9/E496; Supplemental Digital Content, Table 8, available at: http://links.lww.com/JS9/E496).

### Genetic susceptibility and risk of TAA

Two PRS models were constructed: PRS-89, composed of 89 SNPs, and PRS-56, composed of 56 SNPs. The ROC curves further generated suggested that PRS-89 demonstrated better performance compared to PRS-56, with area under the curve (AUC) values of 0.591 versus 0.529 (Supplemental Digital Content, Figure 5, available at: http://links.lww.com/JS9/E496). Therefore, PRS-89 was selected for assessing the genetic risk of TAA.

Generated kernel density plot indicated that participants with incident TAA during the follow-up typically exhibited higher PRS (Supplemental Digital Content, Figure 6, available at: http://links.lww.com/JS9/E496). Further generated RCS curve also showed a positive, linear association between increasing PRS and elevated TAA risk (Supplemental Digital Content, Figure 7, available at: http://links.lww.com/JS9/E496). Individuals with high genetic risk exhibited a 170% increased risk (HR: 2.70; 95% CI: 2.19–3.34; *P* < 0.001) of TAA compared to those with low genetic risk (Supplemental Digital Content, Figure 8, available at: http://links.lww.com/JS9/E496). This suggested a robust dose-response association between genetic susceptibility and TAA risk. The further generated cumulative risk curve indicated that individuals with varying genetic risks exhibited significantly different cumulative risks for TAA (Supplemental Digital Content, Figure 9, available at: http://links.lww.com/JS9/E496).

### Joint effects and interactions of PhenoAgeAccel and genetic risk

**Figure 4.**
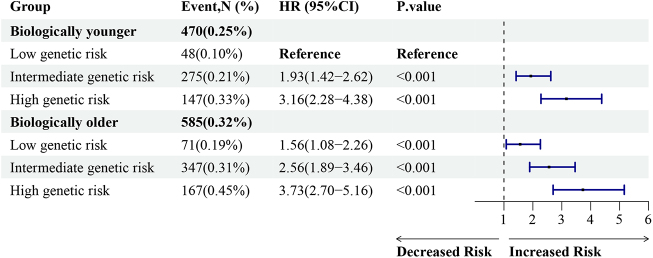
Joint effects of PhenoAgeAccel and genetic risk on the risk of TAA. PhenoAgeAccel: phenotypic age acceleration; PRS: polygenic risk scores; HR: hazard ratio; TAA: thoracic aortic aneurysms.

Joint effects analyses revealed a significant association between accelerated aging and increased risk of incident TAA, across various levels of genetic risk. Compared to biologically younger participants with low genetic risk, biologically older participants with high genetic risk had a 3.73 (HR: 3.73; 95% CI: 2.70–5.16; *P* < 0.001) folds risk of TAA (Fig. 4). Subsequent interaction analyses revealed a significant additive interaction between PhenoAgeAccel and PRS. Among biologically older participants with high genetic risk, the RERI was 0.25 (95% CI: 0.05–0.49; *P* < 0.001), signifying a 0.25 increase in relative excess risk attributable to the additive interaction between PhenoAgeAccel and PRS (Supplemental Digital Content, Table 9, available at: http://links.lww.com/JS9/E496). This interaction accounted for 10% (95% CI: 4–17; *P* = 0.007) of total TAA risk among biologically older participants with high genetic risk.

Genetic risk of individuals was categorized based on their PRS distribution into three categories: low risk (the lowest 20%), median risk (20–80%), and high risk (the top 20%). hazard ratios (HR) and 95% confidence intervals were estimated using Cox proportional hazard models with adjustment for chronological age, sex, ethnicity, education, body mass index, Townsend deprivation index, smoking status, alcohol intake frequency, history diabetes, hypertension, hyperlipidemia, and malignancy.

**Mediation effects of PhenoAgeAccel**

Sex, hypertension and hyperlipidemia have long been regarded significant contributors to incident TAA, those factors also showed significant association with TAA in our study (Supplemental Digital Content, Table 10, available at: http://links.lww.com/JS9/E496). Mediation analyses further revealed that PhenoAgeAccel presents a significant mediating effect in the association between those risk factors and incident TAA (Table 2). The results showed that the associations between gender, hypertension, hyperlipidemia, and TAA could be mediated by PhenoAgeAccel, with mediation rates of 6.18%, 5.06%, and 13.47% respectively.

**Table 2:** Mediation effect of PhenoAgeAccel on the associations between TAA and TAA related factors

	Total effect β (95% CI)	Indirect effect β (95% CI)	Mediation proportion % (95% CI)
Sex, male	2.23 × 10^–3^(1.98 × 10^–3^, 5.43 × 10^–3^)	1.38 × 10^–4^(0.85 × 10^–4^, 3.27 × 10^–4^)	6.18 (3.81, 9.48)
Hypertension	6.25 × 10^–4^(3.21 × 10^–4^, 11.15 × 10^–4^)	3.17 × 10^–5^(1.66 × 10^–5^, 5.12 × 10^–5^)	5.06 (2.71, 8.79)
Hypercholesterolemia	5.18 × 10^–4^(1.33 × 10^–4^, 12.25 × 10^–4^)	6.98 × 10^–5^(4.63 × 10^–5^, 9.55 × 10^–5^)	13.47 (4.20, 59.14)

PhenoAgeAccel: phenotypic age acceleration; TAA: thoracic aorta aneurysm;

Results with 95% confidence intervals were generated from 1000 bootstrap samples. Mediation models were adjusted for chronological age, sex, ethnic, qualifications, BMI, TWI, and comorbidities.

### Enhanced TAA risk prediction by PhenoAgeAccel and genetic risk

Time-dependent ROC analysis was conducted to investigate whether PhenoAgeAccel and genetic risk can assist in identifying high-risk populations for TAA. We constructed three predictive models based on the Cox regression model: the base model, consisting of clinical factors; the PhenoAgeAccel model, which added PhenoAgeAccel to the base model; and the PRS extended model, which added PRS to the PhenoAgeAccel model.

The results indicated that both PhenoAgeAccel model and PRS extended model consistently showed significantly higher AUC values than the base model at all evaluation time points (Supplemental Digital Content, Figure 10, available at: http://links.lww.com/JS9/E496). Similarly, both the PhenoAgeAccel model and the PRS extended model demonstrated a higher C-index than the base model on both the training and the validation cohorts (Supplemental Digital Content, Table 11, available at: http://links.lww.com/JS9/E496). Our results collectively suggested that PhenoAgeAccel and PRS can enhance the prediction of TAA.

## Discussion

In this prospective cohort study, we explored the association between PhenoAgeAccel, a novel aging marker, and risk of TAA. Our findings demonstrated that PhenoAgeAccel was significantly associated with an increased risk of TAA, and it exerted a joint effect with genetic risk on the risk stratification of TAA. Furthermore, this study also indicated that PhenoAgeAccel mediates in the association between various risk factors and the progression of TAA. To the best of our knowledge, it is the first study to combine biological aging and genetic risk for risk stratification of TAA.

Both thoracic and abdominal aortic aneurysms can lead to sudden rupture and massive bleeding, they typically remaining asymptomatic until the onset of fatal complications, making them silent but life-threatening conditions. Standardized screening protocols are currently implemented for the detection of abdominal aortic aneurysms. In the England and Sweden, routine abdominal aortic screening is conducted for men over the age of 65, while in the US, such screening is performed for men over 65 who have a history of smoking^[5,20,21]^. However, in contrast to the standardized screening strategies implemented for the abdominal aorta, there is an absence of a well-defined population screening strategy for the thoracic aorta^[5,22]^. Therefore, identifying high-risk populations for TAA may help provide information for population screening and assist in the development of thoracic aorta screening strategy.

Age is a significant risk factor of aortic disease and also serves as a critical parameter in guiding aortic screening[23]. However, with the deepening of research about aging, the current mainstream view is that both the extent and the rate of aging play crucial factors in the development of diseases[24]. Prior research has established strong association between PhenoAgeAccel, mortality risk and a range of diseases^[25,26]^. However, it is still unclear whether PhenoAgeAccel is associate with the risk of TAA. Based on UK Biobank, we conducted this prospective study to investigate whether PhenoAgeAccel can serve as an effective aging marker for TAA, offering a novel and clinically accessible biomarker for TAA risk assessment. Therefore, future assessments of individuals’ TAA risk should consider not only their chronological age but also their rate of aging, while PhenoAgeAccel provides a convenient method for evaluating the rate of aging. In addition, previous trials on reversing biological aging have shown preliminary inspiring results^[27,28]^. Given the long development period of TAA, PhenoAgeAccel may serve as a potential end-point for preventing TAA through the reversal of biological aging in future clinical trials.

Genetic variants associated with diseases are inherently reliable and stable, and are employed to construct PRS for quantifying individuals’ genetic risk for various diseases^[29–31]^. Genetic risk of diseases reflected by PRS has been demonstrated to interact with environmental factors, and alter individuals’ disease risk jointly^[32–34]^. Since PhenoAgeAccel is primarily influenced by environment and serves as a biomarker for environmental exposures, combining PhenoAgeAccel with genetic risk offers a novel approach to identifying individuals at high risk for TAA[9]. Our findings indicated that the integration of PhenoAgeAccel and PRS can effectively identify populations at high risk of TAA, thereby informing the formulation of screening strategy and primary prevention for TAA. In addition, PhenoAgeAccel exhibited a significant additive interaction with genetic risk, indicating that individuals with accelerated aging and high genetic risk face additional TAA risks. Therefore, assessment of PhenoAgeAccel is crucial in individuals with high genetic risk.

In this study, we incorporated PhenoAgeAccel and genetic risk as predictive indicators into the prediction model for TAA, and then verified that including them will improve the prediction of TAA. However, it is worth noting that, as a long-term condition with a complex disease course, it is difficult to accurately predict TAA by constructing prediction models based solely on these common single-modality clinical indicators. Currently, multimodal clinical prediction models have been widely developed across various disease fields[35]. In the future, incorporating PhenoAgeAccel and genetic risk indicators as modalities into the multimodal models may enable precise prediction of TAA.

Sex, hypertension, and hyperlipidemia are considered as risk factors for TAA[22]. We conducted mediation analyses in this study to and revealed that PhenoAgeAccel significantly mediated the pathogenic process of those risk factors, with a higher mediation proportion observed for hyperlipidemia. This suggested that one of potential mechanism by which hyperlipidemia promoted TAA was through accelerated aging. Therefore, when conducting TAA risk assessment for high-risk populations (such as individuals with hyperlipidemia, males) in the future, PhenoAgeAccel should be given full consideration. To the best of our knowledge, this study represents the first to explore whether biological aging mediates the pathogenic processes of risk factors for TAA.

Based on the large prospective cohort from UK Biobank, we conducted this study to investigate the association between PhenoAgeAccel and risk of TAA, and to integrate PhenoAgeAccel with genetic risk to enhance TAA risk stratification. Nevertheless, several limitations should be noted. First, since UK Biobank primarily consists of healthy individuals of European ancestry, the generalizability of the results to other populations should be approached with caution. Although we have mitigated some of these concerns through subgroup analyses focusing on ancestry and comorbid cardiovascular diseases, potential “healthy/ancestral bias” may still exist. Therefore, caution should be exercised when generalizing the findings to other populations. Second, biochemical markers required for calculating PhenoAgeAccel were only measured at recruitment in the UK Biobank, preventing us from assessing changes in phenotypic age under specific circumstances (such as infection), and the impact of longitudinal changes in PhenoAgeAccel on TAA risk. Third, TAA diagnosis is based on inpatient diagnoses, cause of death, and surgeries undergone, which may introduce reporting and selection biases. Therefore, further research should focus on regular imaging screening for the thoracic aorta, inclusion of diverse populations, and exploring the association between longitudinal changes of PhenoAgeAccel related to TAA.

## Conclusion

PhenoAgeAccel can serve as a novel indicator to identify high-risk individuals of TAA. Combining PhenoAgeAccel with genetic risk will further enhance the risk stratification for TAA, thereby guiding the formulation screening strategy and primary prevention. Given that the calculation of PhenoAgeAccel relies on widely available clinical biochemical markers, it is anticipated to be established as an effective clinical indicator for risk stratification of TAA.

## Data Availability

Data supporting the findings of this study are available at https://ams.ukbiobank.ac.uk/. As these data were used under the current research license, they are not publicly available. However, upon reasonable request and with the permission of the UK Biobank, data can be obtained from Kai Huang (huangk37@mail.sysu.edu.cn).

## References

[1] Tsao CW Aday AW Almarzooq ZI. Heart disease and stroke statistics-2023 update: a report from the American Heart Association. Circulation 2023;147:e93–e621. 36695182 10.1161/CIR.0000000000001123 PMC12135016

[2] Eric MI Ourania P James 3rd HB. 2022 ACC/AHA guideline for the diagnosis and management of aortic disease: a report of the American Heart Association/American College of Cardiology Joint Committee on clinical practice guidelines. Circulation 2022;146:e334–e482. 36322642 10.1161/CIR.0000000000001106 PMC9876736

[3] Mussa FF Horton JD Moridzadeh R Nicholson J Trimarchi S Eagle KA. Acute aortic dissection and intramural hematoma: a systematic review. Jama 2016;316:754–63. 27533160 10.1001/jama.2016.10026

[4] Isselbacher EM Preventza O Hamilton Black Iii J. 2022 ACC/AHA guideline for the diagnosis and management of aortic disease: a report of the American Heart Association/American College of Cardiology Joint Committee on clinical practice guidelines. J Am Coll Cardiol 2022;80:e223–e393. 36334952 10.1016/j.jacc.2022.08.004 PMC9860464

[5] Wanhainen A Van Herzeele I Bastos Goncalves F. Editor’s choice – European Society for Vascular Surgery (ESVS) 2024 clinical practice guidelines on the management of abdominal aorto-iliac artery aneurysms. Eur J Vasc Endovasc Surg 2024;67:192–331. 38307694 10.1016/j.ejvs.2023.11.002

[6] Hibino M Otaki Y Kobeissi E. Blood pressure, hypertension, and the risk of aortic dissection incidence and mortality: results from the J-SCH study, the UK Biobank study, and a meta-analysis of cohort studies. Circulation 2022;145:633–44. 34743557 10.1161/CIRCULATIONAHA.121.056546

[7] Tyrrell DJ Chen J Li BY. Aging alters the aortic proteome in health and thoracic aortic aneurysm. Arterioscler Thromb Vasc Biol 2022;42:1060–76. 35510553 10.1161/ATVBAHA.122.317643 PMC9339483

[8] Liu Z Kuo PL Horvath S Crimmins E Ferrucci L Levine M. A new aging measure captures morbidity and mortality risk across diverse subpopulations from NHANES IV: a cohort study. PLoS Med 2018;15:p.e1002718. 30596641 10.1371/journal.pmed.1002718 PMC6312200

[9] Kuo CL Pilling LC Liu Z Atkins JL Levine ME. Genetic associations for two biological age measures point to distinct aging phenotypes. Aging Cell 2021;20:p.e13376. 34038024 10.1111/acel.13376 PMC8208797

[10] Chen SW Kuo CF Huang YT. Association of family history with incidence and outcomes of aortic dissection. J Am Coll Cardiol 2020;76:1181–92. 32883411 10.1016/j.jacc.2020.07.028

[11] Tcheandjieu C Xiao K Tejeda H. High heritability of ascending aortic diameter and trans-ancestry prediction of thoracic aortic disease. Nat Genet 2022;54:772–82. 35637384 10.1038/s41588-022-01070-7 PMC13102105

[12] Pirruccello JP Chaffin MD Chou EL. Deep learning enables genetic analysis of the human thoracic aorta. Nat Genet 2022;54:40–51. 34837083 10.1038/s41588-021-00962-4 PMC8758523

[13] Wang T Duan W Jia X. Associations of combined phenotypic ageing and genetic risk with incidence of chronic respiratory diseases in the UK Biobank: a prospective cohort study. Eur Respir J 2024;63:2301720. 38061785 10.1183/13993003.01720-2023 PMC10882326

[14] Bycroft C Freeman C Petkova D. The UK Biobank resource with deep phenotyping and genomic data. Nature 2018;562:pp.203-209. 30305743 10.1038/s41586-018-0579-z PMC6786975

[15] Mg ARA Rashid R Kerwan A. Revised strengthening the reporting of cohort, cross-sectional and case-control studies in surgery (STROCSS) guideline: an update for the age of artificial intelligence. Premier Journal of Science 2025;10:100081.

[16] Levine ME Lu AT Quach A. An epigenetic biomarker of aging for lifespan and healthspan. Aging (Albany NY) 2018;10:573–91. 29676998 10.18632/aging.101414 PMC5940111

[17] Jin G Lv J Yang M. Genetic risk, incident gastric cancer, and healthy lifestyle: a meta-analysis of genome-wide association studies and prospective cohort study. Lancet Oncol 2020;21:1378–86. 33002439 10.1016/S1470-2045(20)30460-5

[18] Ye J Wen Y Sun X. Socioeconomic deprivation index is associated with psychiatric disorders: an observational and genome-wide gene-by-environment interaction analysis in the UK Biobank cohort. Biol Psychiatry 2021;89:888–95. 33500177 10.1016/j.biopsych.2020.11.019

[19] SCHOENFELD D. Partial residuals for the proportional hazards regression model. Biometrika 1982;69:239–41.

[20] Abdominal aortic aneurysm screening. nhs.uk https://www.nhs.uk/conditions/abdominal-aortic-aneurysm-screening/

[21] Kelemen M Danesh J Di Angelantonio E. Evaluating the cost-effectiveness of polygenic risk score-stratified screening for abdominal aortic aneurysm. Nat Commun 2024;15:8063. 39277617 10.1038/s41467-024-52452-w PMC11401842

[22] Martin SS Aday AW Almarzooq ZI. 2024 heart disease and stroke statistics: a report of US and global data from the American Heart Association. Circulation 2024;149:e347–e913. 38264914 10.1161/CIR.0000000000001209 PMC12146881

[23] Owens DK Davidson KW Krist AH. Screening for abdominal aortic aneurysm: US preventive services task force recommendation statement. Jama 2019;322:2211–18. 31821437 10.1001/jama.2019.18928

[24] Elliott ML Caspi A Houts RM. Disparities in the pace of biological aging among midlife adults of the same chronological age have implications for future frailty risk and policy. Nat Aging 2021;1:295–308. 33796868 10.1038/s43587-021-00044-4 PMC8009092

[25] Gao X Geng T Jiang M. Accelerated biological aging and risk of depression and anxiety: evidence from 424,299 UK Biobank participants. Nat Commun 2023;14:2277. 37080981 10.1038/s41467-023-38013-7 PMC10119095

[26] Kuo CL Pilling LC Atkins JL. Biological aging predicts vulnerability to COVID-19 severity in UK Biobank participants. J Gerontol A Biol Sci Med Sci 2021;76:e133–e41. 33684206 10.1093/gerona/glab060 PMC7989601

[27] Fleming TR Powers JH. Biomarkers and surrogate endpoints in clinical trials. Stat Med 2012;31:2973–84. 22711298 10.1002/sim.5403 PMC3551627

[28] Fahy GM Brooke RT Watson JP. Reversal of epigenetic aging and immunosenescent trends in humans. Aging Cell 2019;18:p.e13028. 31496122 10.1111/acel.13028 PMC6826138

[29] Nilesh JS Emma B Chris G. Polygenic risk score adds to a clinical risk score in the prediction of cardiovascular disease in a clinical setting. Eur Heart J 2024;45:pp.3152-3160. 38848106 10.1093/eurheartj/ehae342 PMC11379490

[30] Sherafati A Norland K Naderian M Schaid DJ, and Kullo IJ. Polygenic risk and coronary artery disease severity. Circ Genom Precis Med 2024;17:e004470. 39114909 10.1161/CIRCGEN.123.004470 PMC11971913

[31] Sun L Pennells L Kaptoge S. Polygenic risk scores in cardiovascular risk prediction: a cohort study and modelling analyses. PLoS Med 2021;18:p.e1003498. 33444330 10.1371/journal.pmed.1003498 PMC7808664

[32] Cui F Sun Y Xie J. Air pollutants, genetic susceptibility and risk of incident idiopathic pulmonary fibrosis. Eur Respir J 2023;61:2200777. 36137588 10.1183/13993003.00777-2022

[33] Wang L Xie J Hu Y Tian Y. Air pollution and risk of chronic obstructed pulmonary disease: the modifying effect of genetic susceptibility and lifestyle. EBioMedicine 2022;79:103994. 35417845 10.1016/j.ebiom.2022.103994 PMC9018147

[34] Zeng L Wu Z Yang J Zhou Y Chen R. Association of genetic risk and lifestyle with pancreatic cancer and their age dependency: a large prospective cohort study in the UK Biobank. BMC Med 2023;21:489. 38066552 10.1186/s12916-023-03202-0 PMC10709905

[35] Zhang K Zhou R Adhikarla E. A generalist vision-language foundation model for diverse biomedical tasks. Nat Med 2024;30:3129–41. 39112796 10.1038/s41591-024-03185-2 PMC12581140

